# The impact of social network change and health decline: a qualitative study on experiences of older adults who are ageing in place

**DOI:** 10.1186/s12877-021-02385-6

**Published:** 2021-09-04

**Authors:** Willeke Vos-den Ouden, Leonieke van Boekel, Meriam Janssen, Roger Leenders, Katrien Luijkx

**Affiliations:** 1 Department Tranzo, Tilburg University, Tilburg School of Social and Behavioral Sciences, P.O. Box 90153, 5000 LE Tilburg, The Netherlands; 2 Department Organization Studies, Tilburg University, Tilburg School of Social and Behavioral Sciences, P.O. Box 90153, 5000 LE Tilburg, The Netherlands; 3 Jheronimus Academy of Data Science, St. Janssingel 92, 5211 DA ‘s-Hertogenbosch, The Netherlands

**Keywords:** Social network change, Impact, Experiences, Ageing in place

## Abstract

**Background:**

Older adults prefer to age in place. Social network change and health decline challenge ageing in place, as stressors that make age-related advantages disappear. The aim of this study was to explore social network change and health decline and its impact on older adults who are ageing in place.

**Method:**

In-depth interviews (*n* = 16) were conducted with older adults who were ageing in place and who were experiencing health decline and social network change. Procedures for grounded theory building were followed to analyse the interviews with respondents who were discharged from the hospital less than 4 months ago (*n* = 7). Narrative analysis was conducted to reach a deeper understanding of the expected complexity of experiences of this targeted sample.

**Results:**

Results encompass a typology with four types of impact: A. Sneak preview of old age, B. Disruptive transition into old age, C. Drastically ageing, and D. Steadily ageing. Additionally, indications were found that older adults should be able to move along the four types of impact and ideally could end up in quartile D, experiencing little or no impact at all (anymore).

**Conclusion:**

The results present an optimistic view on the possibilities of older adults to continue ageing in place despite experiencing unavoidable and uncontrollable stressors in life. Also, the results provide leads for practice, to develop an action perspective for home care nurses and gerontological social workers to determine and reduce the impact of social network change and health decline on older adults who are ageing in place. Suggestions for further research would be to unravel how to detect temporal setbacks in successful ageing in place.

**Supplementary Information:**

The online version contains supplementary material available at 10.1186/s12877-021-02385-6.

## Background

Older adults prefer to age in place [1–3] or are forced to age in place in low- and middle-income countries [4, 5]. Governments of welfare states promote ageing in place to prevent older adults from rather expensive institutionalisation and to deflect healthcare costs [6]. Social networks and sufficient health are preconditions for older adults to age in place [7]. Social networks are a source of support [8, 9] and contribute to health and well-being [10–18] although the composition of the social networks culturally diverges [19, 20], as do norms on informal care giving [4, 21]. In welfare states, good health precludes older adults from nursing home admission [22–24]. Older adults’ social networks and health are likely to change though, which challenges ageing in place. Friends and loved ones may be lost, caregivers may enter the social network to support older adults [25–28], and health decline is likely to occur [29, 30].

In the literature, many results have been published regarding the impact of social network change and health decline on older adults who are ageing in place in Western societies. Studies on socio-emotional trajectories throughout the life-span [9, 31–33] indicate that older adults report relatively high levels of emotional well-being despite changing and declining social networks [34, 35]. Moreover, social networks continue to play a positive role in well-being, despite their decline [36, 37]. Older adults appear to select social partners and activities that fit – and are continuously adapted to – their changing life goals [31, 38]. Also, older adults become more experienced in how they can regulate their emotions and social lives [33]. However, these general age-related advantages might disappear when unavoidable and uncontrollable stressors are experienced [34, 39], such as social network changes and health decline.

Social network changes and health decline may be perceived as unavoidable and uncontrollable (temporarily) stressors. These stressors have the potential to make age-related advantages of social network change disappear and have a negative impact on older adults, which compromises ageing in place. In this study, we aim to add to the literature a more comprehensive understanding of these stressors. Therefore, we explore the impact of health decline and social network changes on older adults by appreciating older adults as experts on the subject and by using a qualitative research design to understand the complexity of older adults’ personal experiences more thoroughly [39]. The following research questions will be answered: 1) What are older adults’ experiences with health decline and social network change while ageing in place? 2) What is the impact of these experiences on older adults who are ageing in place? 3) Why do experiences with health decline and social network change impact older adults who are ageing in place, or not?

## Method

### Study design and setting

An exploratory qualitative research design was followed to explore the experiences of health decline and social network change of older adults who are ageing in place. A brief interview protocol allowed the respondents to narrate the subject freely, leaving room for their own subject matters and main concerns [40]. The study took part in the southern region of the Netherlands, in the homes of the respondents, in the presence of only the respondent and the interviewer to ensure confidentiality [41]. Appendix A includes a report on the Standards for Reporting Qualitative Research to account for the quality criteria that are followed [42].

### Participants and recruitment

The in-depth interviews were conducted with older adults (*n* = 16) aged > 65, who were recruited through four home care organisations from the networks of the Academic Collaborative Centre Elderly (ACC) [43] by home care nurses who volunteered to participate in the selection procedure. The inclusion criteria were ‘the older adult is experiencing acute or chronic health decline’ and ‘the home care nurse notices social network change within the life of the older adult’. The nurses delivered care to the older adults, and if their client was interested in participation and fitted the inclusion criteria, then the home care nurse handed over an information set. The information set contained information on the research topic and on how to participate and an informed consent form. After returning the forms, a researcher (WV or MJ) contacted the respondent and made an appointment for the interview. After a period of 1 year, the enrolment of older adults by home care nurses lingered and eventually stopped, despite several reminders. The researchers then chose to divide the sample, which fits the inclusion criteria, into two subgroups: a) older adults who were admitted to the hospital recently, who returned home and received home care afterwards, and b) older adults who lost their spouses (either because of death or because of moving into a nursing home) after a period of illness in which their spouse received home care. This choice provided the opportunity to analyse a targeted sample of older adults who are ageing in place with specific experiences and to explore in detail the similarities and differences between respondents [44]. The present study included the respondents of subgroup *a*, who were admitted to the hospital less than 4 months ago and who continued ageing in place afterwards (*n* = 7). The respondents received a small present after the interview when they were thanked by the researchers.

### Data collection

Six interviews were held by the first author (WV), who was a PhD student and was experienced in interview techniques as a team coach and health care consultant. One interview was held by the third author (MJ), who has a PhD in qualitative research and who has worked as a senior researcher at the ACC. The interviews were guided by a protocol that consisted of two parts. The first part was an introduction of the interviewer, an overview of the research goals and a check of the informed consent form. In the second part, the audiotape was started after the respondent gave his or her permission. Subsequently, two questions were asked: “From [name nurse] I heard you experienced health decline recently. Can you tell me more about that period?” and “Do you perceive your life has changed since your health decline?” Additionally, a set of supporting questions was asked, on the one hand to draw out experiences – “How did you feel about that?” and “How did you deal with that?” – and on the other hand, to guide the narrative toward social network changes – “Do you still do the same things as before your health decline?” and “With whom?” The interviews lasted between 60 and 120 min and ended when information saturation appeared to be reached. The interviews were audiotaped and transcribed verbatim. Original files were stored in accordance with the data management protocols of Tilburg University and transcripts were anonimised by the first author before data analysis. Data (*n* = 16) were collected between December 2018 and November 2019.

### Data analysis

The data analysis followed procedures for grounded theory building [45] because of its usefulness for understanding specific phenomena within processes, such as experiences of social network change and health decline while ageing in place [46]. Additional efforts were made to understand the experiences of each respondent by considering each in-depth interview as a narrative. It is through narratives that we make sense of our social worlds [47]. Therefore, analysing the narratives of each respondent helped us to go beyond understanding the experiences and examine how respondents make sense of their experiences themselves, in order to reach a deeper understanding of the expected complexity of personal experiences [44, 47, 48].

First, the interview with respondent 1 was open coded in Atlas.ti 8, and the researchers discussed these codes until consensus was reached (WV, MJ, LB). For example, a text fragment of respondent 1 -“the assistant of the general practitioner did nothing too, just nothing, nothing” was coded with ‘abandoned by professionals’. Second, the coded interview of respondent 1 was read again by the researchers (WV, MJ, LB) and a listening guide (Table 1) was used to guide the reading, analysis, and discussion of the narrative of all three researchers and thus to enhance reliability. The researchers answered the questions of the listening guide and discussed their answers until they reached consensus (WV, MJ, and LB). Third, the next interviews (2–3) were coded openly and discussed (WV, MJ, LB). After consensus was reached, the code list in Atlas.ti was slightly adapted, and the remaining interviews (4–7) were also open coded (WV). For example, open codes were: ‘quality of contact with doctor’, ‘abandoned by professionals’, ‘needing more’, ‘feeling unheard/unseen’. Parallel to the coding process, the remaining interviews (2–7) were read again, analysed and discussed with the Listening Guide. Discussions were held in duos – three interviews were discussed by WV and MJ and three by WV and LB – until consensus was reached. As part of the discussions, the coding of each interview was also evaluated, and codes were grouped into themes. For example, the previously mentioned codes became part of the overarching theme ‘preferring permanent medical attention and involvement’. The codebook was added as an additional file. Fourth, in order to construct the meaning of the words of the respondents, comments were added to text fragments in Atlas.ti within each interview (WV). The comments differentiated between *what* the respondent said, *how* he or she said this, and *why* at that moment in the interview, and how the researchers *interpret* this fragment in relation to answering the research questions. The comments were read by the other researchers and were discussed in the same duos until consensus was reached about the meaning of the words of the respondents (WV and MJ; WV and LB). For example, respondent 6 told how her children took care of her after her hospital admission (*what*) and she used this fragment as an introduction (*why*) to tell how often her children still visited her and how much she enjoys it (*how*) as she finishes this fragment making her point (*interpret*) that she (hospitable, cheerful, optimistic) and her husband (pessimistic, dominant) are very different persons. Finally, a summary was made of all seven interviews, with the open codes used, the themes, the first-discussion remarks, the second-discussion comments, and a summarised interpretation of the researchers. The summaries and the preliminary versions of this paper were discussed with the researchers and the supervisors (WV, LB, MJ, KL, RL) to answer the research questions.

**Table 1:** Questions that were used to discuss and analyse respondents’ narratives. Questions were based on the four required ‘readings’, which are needed to operationalise a narrated subject, also referred to as ‘the Listening guide’ (Doucet and Mauthner 2008)

1. Discussing the narrative	a. What is going on in the life of this respondent?
	b. What appear to be important words, themes, events, contradictions, persons in their lives or chronologies?
	c. What would you code differently?
2. Discussing people in the narrative	d. How does the respondent speak about herself/himself?
	e. How does the respondent speak about other people? Which identity does she/he give them?
3. Discussing relations in the narrative	f. Which relations are part of the narrative?
	g. How does the respondent speak about these relations?
4. Discussing structures and processes in the narrative	What is indicated about:
	h. … different life stages and events that happened in these life stages?
	i. ... health decline?
	j. ... social network change?

## Results

All seven respondents were admitted to the hospital less than 4 months before the interview and, afterwards, continued to age in place with the support of home care. All respondents were alone in their homes during the interview. Table 2 displays the gender and the health and social statuses of the respondents.

**Table 2:** Respondents’ gender, health status, and social status at the moment of the interview

Respondent		Health status		Social status		
Nr.	Gender	Health decline	Cause of hospital admission	Marital status	Children	Most important contacts
1	F	acute	fall	unknown	yes	friends
2	M	chronic	Parkinson’s disease	widowed	yes	friends
3	M	acute	stroke	widowed	yes	friends
4	F	chronic	cancer	unknown (1)/ widowed (2)	yes	family
5	M	chronic	cancer	single	no	friends
6	F	acute	feeling unwell	married	yes	family
7	M	chronic	cancer	married	yes	family

After asking the first question and sometimes even before the interviewers had unpacked their materials, respondents started to tell their stories. Respondents’ initial narratives spanned periods that diverged from solely telling about the present, concerning the period of hospital admission (1, 6), to starting their stories with major life events, concerning their early childhood (5) or young adulthood (4). Two respondents’ stories began with the loss of their wives, over a decade ago (2, 3), and one respondent’s initial story spanned a decade of health decline (7). In this paper, the results focus on the changes in the period of ‘old age’ in the respondents’ lives. The respondents defined ‘old age’ as the period in life in which the respondent had finished his or her working life or the working life of his or her spouse and which is sometimes (1, 2, 3, 5, 6) explicitly marked by the respondent with a life event (i.e. loss of spouse, retirement of spouse, illness diagnosis). In Table 3, the health changes and social network changes of each respondent are reported.

**Table 3:** Respondents’ health change and social network change in the life stage of ‘old age’

Respondent		Health change		Social network change		
Nr.	Gender	Health decline before hospital admission - concerning the life stage of ‘old age’	Health decline after hospital admission - compared to health status before admission	Addition of people	Loss of people	Change of existing relations
1	F	Regular aging with negligible health decline	Small	Home care professionals	No	Receiving support from a few close friends
2	M	Large decline due to chronic illness in past decade	Small	Home care professionals	Wife, Friends	Receiving more support from son and daughter-in-law
3	M	Regular aging with negligible health decline	Large	Home care professionals and rehabilitation professionals	Wife, Friends	Receiving more support from daughter and granddaughter
4	F	Large decline due to chronic illness in past two years	Small	Home care professionals	Family, Friends	Only reciprocal relations with family members and friends remained
5	M	Regular aging with negligible health decline	Large	Home care professionals, biological sister	Friends	More intense contacts with friends/neighbours; only superficial contact left with biological sister
6	F	Regular aging with negligible health decline	Small	Home care professionals	Acquaintances	Receiving more support from daughters
7	M	Large decline due to chronic illness in past two years	Large	Home care professionals	Friends/ Acquaintances	Receiving more support from (grand)children; less contact with friends/acquaintances

Moreover, respondents described various situations and defining moments that occurred in their life stage of ‘old age’. Below, the emerging themes on experiences with health decline are mentioned: “temporarily losing yourself,” “preferring permanent medical attention and involvement,” “home (bitter) sweet home,” “being aware of a permanent setback,” “adapting oneself and continuing life.” Respondents also narrated their experiences of social network change, and below, the emerging themes were presented: “regretting social network losses,” “gratitude for support,” and “persisting social engagement.”

### Experiences with health decline

#### Temporarily losing yourself

Respondents hardly had any memories of their stay in the hospital or missed several days of the admission from their memory (1, 2, 3, 5, 6). They recalled that they hallucinated (1, 3), they failed to recognise the doctor (1), or they were conscious but could not participate in a conversation (2, 3, 5, 6). This made them feel anxious and helpless, specifically – according to respondent 1 – because she was not even able to formulate a request for help anymore:

*“And I have been very anxious, I did not recognize myself anymore, because I’m not such a fearful type of woman, not at all.”*

#### Preferring permanent medical attention and involvement

Respondents mentioned specific moments of contact they had with their doctors (1, 2, 3, 4, 5, 7). Respondent 2 expressed great faith in his neurologist – as *“he always tries to make the most of it”* – and respondent 1 appreciated when her physician talked to her as an equal person. However, respondents also mentioned that their doctors were hardly available for a conversation during their period of health decline (1, 5), and retrospectively, they expected more information or insights on their treatment (1, 4, 5). Once home again, when medical involvement had been terminated or scaled back, respondents felt abandoned by their doctors (1, 4, 5), insecure about their further rehabilitation (1, 3), and that they needed more medical involvement and support (1, 3, 5) to get well again. Respondent 3 told about his further rehabilitation at home:

*“There, in the rehabilitation centre, I was Mr. (name). And how far is Mr. (name)? There was overview, you got an overview. So, you could ask: what about the future, how do you see the future? And then you got an answer that was supported by all therapists. ( …*). *But now, here [WV: at home], the speech therapist can tell me what she thinks, but it does not bring me anywhere if my mobility is not alright yet!”*

#### Home (bitter) sweet home

All respondents received support, over a longer period or since the hospital admission, from family members (2, 3, 4, 6, 7), from home care or at-home rehabilitation professionals (3, 5, 6, 7), or from friends or acquaintances (1, 3, 4, 5). During the recent period of health decline, they were surprised by unexpected visits, postcards, and other expressions of attention (1, 2, 4, 5, 6), and in general, they were satisfied with the support they received (1, 2, 5, 6, 7). However, respondent 3 experienced stress because of the large number of professionals that suddenly entered his home after hospital admission. He felt himself forced to keep an agenda and schedule the appointments he had to make. It made him feel robbed of his freedom. Respondents 1, 3, and 5 felt that they needed more support with household activities when they arrived back home. They had to take care of more issues than before hospital admission – medication, appointments, arranging help – but were less able to do so. Feelings of anxiety and helplessness struck them. Although respondents mentioned that it is nice to be home again (1, 5, 6), they expressed that it is not easy to be a patient in your own home (1, 3, 5) and it takes a certain amount of time to create order and oversight at home again (1, 2, 3). Respondent 5 told the interviewer how he came home, alone, on Christmas Eve, after a hospital admission of a week:

*“And that’s what they send me home with, with the message that I had cancer and that I would get surgery soon. Well, I can tell you, I have never had such a rotten Christmas like then. All alone, I was in the hospital all week, and when I came home the shops were closed. ( …*) *So I sat here alone, with a piece of dry bread, I had no groceries in my home. I have never had such a horrible Christmas.”*

#### Being aware of a permanent setback

After a period of health decline, respondents stated that they kept having a hard time overseeing their home situation (1, 3). They perceived that the recent health decline had affected their abilities to structure their daily lives (1, 2, 3). Respondents discovered that a full recovery might not be feasible (1, 5, 6, 7) and that some physical constraints may remain (2, 3, 5, 6, 7). This raised doubts and insecurities about the recovery that might be expected (1, 3, 4, 5) and on the extent to which they would fully recover into their old selves anyway (1, 2, 4, 5, 7). Respondent 7 told that he re-organised his medication intake for the progressive illness he suffers, as to lose less energy on getting up in the morning, which should enable him to regain his physical condition hopefully partly after his hospital admission:

*“And yesterday, I tried my bicycle for the first time again, but that was a disappointment. My physical condition is not good enough yet. (…) So, that’s what I start with today: every day some practicing on my hometrainer to strengthen my legs, because even when walking I notice it’s not what it should be.*

#### Adapting oneself and continuing life

Irrespective of a chronic or an acute health change, respondents reported that their lives had changed (2, 3, 5, 6, 7) in a sense that they had become more dependent on others (3, 4, 5, 7) and that their radius of action had become smaller (all), whether or not the change was temporary (3). In the end, they still felt they had to be self-reliant (1, 2, 5, 7) and had to accept their situation (all); although, they ensured the interviewers that they still managed themselves quite well (1, 2, 3, 5). Respondent 2 answered, to the question if he still goes out sometimes, despite impediments:

*“No, because I can’t do it anymore, I cannot … if I go out in the afternoon, then I can’t go in the evening … I don’t do it anymore. On a birthday [WV: where it is noisy], at first I participated, if you know what I mean, and now I just sit there and well, sometimes someone comes to sit next to me to talk ( …) and I don’t drink anymore, so … Well, I go in the mornings, a couple of hours and then … then I just go home again. No, I don’t care, it’s not bothering me.”*

### Experiences with social network change

#### Regretting social network losses

All respondents mentioned the contacts they had with family, friends, neighbours, acquaintances, and the people with whom they shared a hobby or a field of interest. They expressed happiness with rich social lives (1, 4, 6) or sadness because of the loss of it (2), whereas spontaneous visitors and gatherings were referenced as examples of a rich social life (1, 2, 6). However, the rich social lives appeared to have changed, sometimes rapidly and sometimes slowly, due to a decreasing radius of action due to health decline (1, 2, 3, 7). Social contacts were lost whereas new ones remained absent (2, 3, 4). The absence of contacts felt like a limitation of being oneself (2, 3) and as a gap (5). Holidays and day trips were gone, and hobbies were not accessible or reachable anymore (1, 2, 3, 7). Remaining social contacts diluted and faded out (2, 4, 5, 6). Respondent 2 reflected:

*“Well, it [WV: his social network] keeps on narrowing, right? I have to say, it becomes … it evolves like that, very slowly. If you think back, then you recognize what you used to do, during the day en then you see, well … now you do almost nothing, as a matter of fact.”*

#### Gratitude for support

In periods of health decline, respondents were surprised by the attention of close and more peripheral contacts and were grateful for the support they received from family, friends, and professionals (1, 3, 4, 6, 7). However, it appeared to be important for respondents that supporters tried to understand their situation. It made them feel heard and seen (3, 5, 6). Moreover, being able to return a favour to helpers made respondents feel as if they were still needed somewhere (1, 6, 7). Respondent 3 briefly illustrated his situation of being in the middle of social losses on the one hand and being grateful for the received help on the other. He felt frustrated because he was restricted in his freedom, by which he meant that he cannot take his time to shop anymore, lingering and meeting acquaintances from his old village, and having small talk.

*‘*. *.. well the groceries are not a problem, I’m sure they’ll arrive. But everything around it, well that’s gone!’*.

#### Persisting social engagement

Respondents claimed to be social people (1, 2, 5) and hospitable (6). Respondents mentioned that they liked to go out (1, 2, 3) and that they were willing to continue social participation (5, 6). Spontaneous meetings and visits were considered as a substantiation of wealthy living (1, 2, 6). Respondent 6 was proud of her hospitality and very pleased with the amounts of people it still brought to her home:

*“Well, they [WV: her eight children and their families] come home a lot. Last Saturday, it was remarkable, because they had … , they come to have a coffee then, sometimes all of them, sometimes three or four of them and then there was –what a coincidence- the one with the baby, the partner of my grandson … well, I’m having the time of my life at that moment!”*

In Table 4, the prevailing themes per respondent on health decline and social network change are summarised.

**Table 4:** Prevailing themes per respondent on health decline and social network change

Nr	Gender	Prevailing themeshealth decline	Prevailing themes social network change
1	F	Temporarily losing yourself	Gratitude for support
		Preferring permanent medical attention and involvement	Persisting social engagement
2	M	Preferring permanent medical attention and involvement	Regretting social network losses
		Adapting oneself and continuing life	Gratitude for support
			Persisting social engagement
3	M	Preferring permanent medical attention and involvement	Regretting (temporary?) social network losses
		Home (bitter) sweet home	Gratitude for support
			Persisting social engagement
4	F	Adapting oneself and continuing life (negatively)	Regretting social network losses (earlier age)
			Gratitude for support
5	M	Preferring permanent medical attention and involvement	Regretting social network losses
		Home (bitter) sweet home	Gratitude for support
		Being aware of a permanent setback	Persisting social engagement
6	F	Adapting oneself and continuing life (positively)	Gratitude for support
			Persisting social engagement
7	M	Home (bitter) sweet home	Regretting social network losses
		Being aware of a permanent setback	Gratitude for support
		Adapting oneself and continuing life (negatively)	

### Impact of health decline and social network change

Although experiences regarding health decline and social network change appeared to be generally present in the interviews with the respondents, the impact of these changes appeared to differ widely between respondents.

#### Impact of health decline

Respondents 1, 3, and 5 were overwhelmed by the health decline that struck them for the first time in their lives. They were interviewed right in the middle of this life altering experience (3, 5) or afterwards, when life had taken its course again (1). Narratives with emotional statements expressed the impact of the recent health decline. Respondents 2, 4, 6, and 7 were already familiar with health decline or were gradually ageing. The recent hospital admission was only a small part of their narratives and appeared to have had hardly any impact. Respondents 1, 3 and 5 repeatedly got agitated while telling the story on their health decline, e.g. respondent 1 about the period that she stayed in the care hotel:

*“When can I go home then?’, ‘Well, that’s what you have to decide yourself’, Oh, I cannot hear the word “help request” anymore! ‘You have to request for help yourself.’ I said: ‘now what?’ ‘You are the spider in your own web, you have to arrange things yourself’. I said: ‘you know what I am?! I am a spider that dangles to only one thread and I can only try to not break that tread, that’s all!!’*

#### Impact of social network change

A high impact of social network change was found when loved ones (i.e. family or friends) were lost permanently or temporarily. Respondents were recently isolated from their friends (3), had to deal with a cooled off contact with a sole biological family member (5), or had drifted away from earlier social lives in the past decade (2, 7). Narratives of these respondents were permeated with frustration, melancholy, and sadness with respect to this topic. On the contrary, social network change hardly appeared to have impacted other respondents (1, 4, and 6). They had not lost anyone (1) or lost a few acquaintances but felt little regret about it (6) or lost family members in earlier life, as part of a complicated family history, but appeared to have accepted that (4). Respondents 2, 3, 5 and 7 narrated the profound impact of the changes in their social networks, e.g. respondent 7. He spoke extensively regarding, what he called, ‘social work’ with war veterans. Several stories passed by on his activities for this group, on the trips to foreign countries for them and the gratefulness he received from the veterans and, later, from their descendants. He sight:

*“Well, it has become a ‘sitting’ life now. In contrast to earlier days, because there were always lots of activities, even if I was retired.”*

**Fig. 1:**
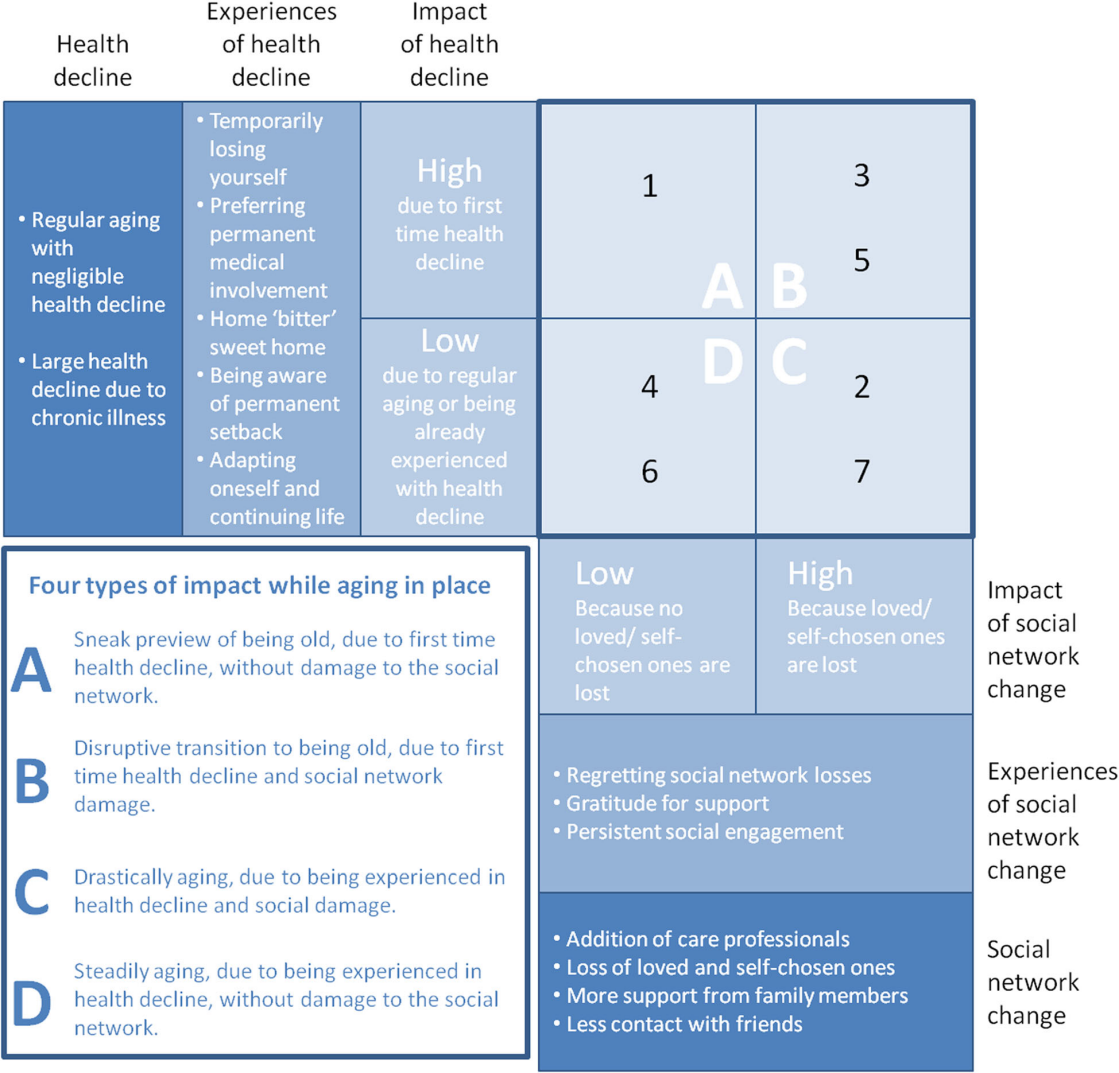
A preliminary typology of impact while ageing in place. The typology comes along two dimensions of impact that we explored i.e. health decline and of social network change

The interviews with the seven respondents were plotted into a table along dimensions of health decline and social network change. The impact of experiences on both dimensions were qualified as ‘high’ or ‘low’ for every respondent. This exercise generated a preliminary typology of impact while ageing in place and experiencing social network change and health decline (Fig. 1).

#### The typology contains the following four types of impact

A. Sneak preview of being old, due to first time health decline, but without damage to the social network (yet).
B. Disruptive transition to being old, due to first time health decline and damage to the social network.
C. Drastically ageing, due to being experienced in health decline and social network damage.
D. Steadily ageing, due to being experienced in health decline or due to regular ageing, but without damage to the social network.

### Occurrence and height of impact

In addition to how experiences of health decline and social network change impact older adults, we found indications in the narratives regarding why respondents experienced different impacts despite similar experiences of health decline and social network change. The first indication is that determining impact, based on an interview, appeared to be equivalent to making a snapshot of someone’s life. Moreover, the snapshot is taken during a period of changes. The second indication is that, despite having experienced similar events, impact was only present when preferences, values, and needs were not met during these events.

#### Determining impact is equivalent to making a snapshot

Interviews appeared to be a single moment within a life course. Respondents carried a lifelong history with them. The histories contained impactful, or even traumatic life events (2, 3, 4, 5, 6, 7), sometimes even from early childhood on. However, respondents not only narrated their past and present, but they also mentioned their future. They told the interviewers to persevere and carry on, and they looked forward to their near future (1, 3, 4, 5, 6), to recovery, to move to other living arrangements, to an uncertain future, or even to a self-chosen moment of death. Experiences of social network change and health decline were placed within an historic context and sometimes made histories revive. Respondent 1 looked back at her temporary period of health decline and was relieved that her social life had retaken its regular course, whereas respondents 3 and 5 were caught right in the middle of a period of health decline and they were anxious to get their health and social life back soon. Respondents 2 and 7 did not have that prospect. They were at a moment in their life courses in which their health could only get worse and, parallel to that, their social lives too. For example, the narratives of respondents 2 and 7 mainly contained pleasant stories and anecdotes about past times, which contrasted sharply with their current daily lives. Therefore, making two snapshots within a certain time span could result in different types of impact. This would mean that respondents should be able to move along the typology.

#### Impact is determined when preferences, values, and needs are not met

Preferences varied between respondents; for example, some respondents preferred community-based and cultural activities – such as going to the theatre, joining a senior’s orchestra, or organising a city-event (1, 3, 7), − while others preferred social encounters with friends, outdoors (2, 5), or with family at home (6). Also, variety occurred in the extent to which preferences could still be fulfilled with the resources they had left after health decline and social network change. For example, respondent 1 was used to participating in cultural activities, and despite her health decline, she was still able to organise a visit to a piano recital with friends, including transport and special seats for visitors in a wheelchair. On the other hand, respondent 2 preferred to go out with friends, playing cards until dawn and grabbing a beer downtown. However, he appeared to be unable to maintain these outdoor contacts with his friends due to his ongoing health decline, and he also appeared to find it hard to talk about the difficulties in his changing life and that of his friends. Moreover, his health decline hindered him from going to crowded places with loud noises and make new friends.

Next to preferences, values and needs in respect to social encounters also varied between respondents. For example, autonomy and self-management (1) and independence and freedom (3) were prevailing values that could not be met during a period of health decline. This made respondents 1 and 3 feel frustrated and anxious. For respondent 5, being heard and seen was very important but not realised because his close friends could not fill the gap of his biological family. This made him feel alone anyway, but since his health decline, even more than ever because he could not share his story with a significant other. Respondents 2 and 7 revealed that the social contacts they presently missed served purposes of hedonism (2) and acknowledgement (7), which prompted feelings of melancholy. In contrast, respondents 4 and 6 valued reciprocity of relations highly, and they appeared to still be able to maintain reciprocal relations. They appeared to still feel needed: one constantly invited people into her home for coffee and a listening ear (6) and another expressed satisfaction on the few reciprocal contacts she had left despite a loss of family and friends in the past (4). This would mean that health decline and social network change do not automatically have impact (quartile D). Impact occurs only when preferences, values, and needs are not met (quartiles A, B, and C).

## Discussion

In exploring social network change and health decline and the impact on older adults who are ageing in place, we found experiences of health decline (i.e. temporarily losing yourself, preferring medical involvement, home (bitter) sweet home, being aware of a permanent setback, adapting and continuing life) and experiences of social network change (i.e. regret, gratitude, and persisting social engagement). However, despite similar experiences of comparable events, the impact appeared to vary greatly between respondents. Plotting the interviews into a table and qualifying the impact as high or low resulted in a preliminary typology of ‘impact while ageing in place’. The four types of impact that we distinguished were A. Sneak preview of old age, B. Disruptive transition into old age, C. Drastically ageing, and D. Steadily ageing. Additionally, we found indications of why respondents experienced different impacts despite similar experiences of health decline and social network change. We found that determining impact is equivalent to making a snapshot, which means that older adults could move along the typology when making multiple snapshots over time. Finally, we found that impact was determined when preferences, values, and needs were not met, which means that preferences, values, and needs were met in quartile D and were not met in the other quartiles.

### Results in a broader perspective

Our exploration of experiences of social network change and health decline and its impact on older adults who are ageing place is informative on personal differences despite similar experiences but leaves older adults underexposed as ‘active agents’. For that matter, the literature indicates that older adult’s characteristics, e.g. self-esteem, mastery and self-efficacy, can contribute as coping resources in regaining wellbeing after persistent health decline [49]. A more recent study indicated that active attempts at emotional expression and processing in response to stressful circumstances (i.e. emotional approach coping) appeared to be protective against declining health [50]. To reach a comprehensive understanding on the subject it could be recommended not only to appreciate older adults as experts, but also to consider them as active agents. Furthermore, the results of the present study provide a clear view on moments in the life of older adults in which ageing in place is being challenged. However, it should be taken into consideration that the prevalence of this situation is restricted to welfare states and urban regions in which alternatives for ageing in place and traditional family care are present and accessible. Moreover, if the results are placed within the perspective of the Covid19 epidemic, then it could be reckoned that social network changes due to social restriction measures will have increased, as well as the number of older adults that experience a negative impact, which was supported and nuanced by recent evidence on this subject [51–54].

### Results in relation to the academic literature

Firstly, the results of the present study appear to confirm the premise that social network change and health decline are unavoidable and uncontrollable stressors that disrupt successful ageing in place (Sims, Hogan [39]. The health decline appeared to play a determining role in the lives of the respondents, temporarily (1, 3), as a surprise (1, 3, 6) or consistently (2, 4, 5, 7), in a way that it changed their social networks and their lives in general. Respondents in quartiles A, B, and C of our typology experienced a negative impact of health decline and/or social network change. However, a disruption of successful ageing appeared not to be an inevitable effect of the stressors, considering that some respondents apparently found ways to fulfil their preferences and needs and continued successful ageing in place without noteworthy impact. This finding resonates with the literature on resilience among older adults. For instance, different factors are found to affect the resilience of older adults, such as income and mental health [55], emotional regulation ability and problem solving [56] or having adaptive coping styles, optimism and hopefulness, positive emotions, social support and community involvement, performing activities of daily living (ADLs), independence, and being physically active [57]. We found indications of differences between respondents that resonate with these factors that affect resilience; although, we did not have enough evidence to confirm these findings.

Secondly, our results corroborate with the literature on experiences of transitional care and of daily life after hospital discharge. The experiences we found on health decline (e.g. preferring medical involvement, home (bitter) sweet home, being aware of permanent setback, and adapting and continuing life) reflect a process of negotiation and navigation of (in) dependence after health decline. According to a meta-synthesis of qualitative studies, older adults are likely to face challenges in care coordination, in managing the health and social consequences of hospital admission (such as pain and fatigue, but also feelings of loneliness), and in finding their way throughout life again [58]. Another meta-summary indicates that, in daily life after hospital discharge, older adults experience a lack of information, confusion about medication, and a lack of involvement in their treatment and in decision making. Moreover, this meta-summary also resonates with the experiences we found on social network change (regret, gratitude) as they state that older adults feel the loss of independence, feel a large dependence on family and friends, and appear to be aware of the effort it requires of these informal care givers. In sum, regaining a balance between dependence and independence and regaining (or maintaining) one’s own social life after hospital discharge are analogous findings to our study.

Thirdly, our results – indicating that impact of health decline and social network change varies between older adults – are in line with findings on the social diversity of older adults who are ageing in place. Literature not only indicates that a diversity in social needs is culturally and individually determined [59] and that, for example, action orientation and coping strategies are distinguishing factors between socially isolated older adults [60]. It was also discussed that subjective factors, such as life experiences, family dynamics, and long-term patterns of socialisation, should be considered when trying to understand older adults’ (small) social networks as a personal lived experience [61]. In quantitative research, multidimensional and longitudinal data were deployed to be able to distinguish subgroups of older adults who are more or less successfully ageing in place [62].

### Results in a future perspective

This study has implications for further research as well as for practice. A suggestion for future research would be to unravel how to detect temporal setbacks in outcomes of social health, well-being, and successful ageing among older adults who are ageing in place. This would contribute to a more comprehensive understanding of how resilience and the ability to adapt and to self-manage among older adults may be enhanced [14, 57, 63]. Suggestions for the practice of home care nurses and gerontological social workers [64] would be to use our typology to develop an action perspective on determining the impact of health decline and social network change in older adults who are ageing in place and on finding ways to reduce that impact.

### Strengths and limitations

A limitation of this study is the relatively small number of respondents that we interviewed. The home care nurses who recruited participants were all confronted with a personnel shortage and increasing demands. In fact, actively recruiting participants for this study appeared time-consuming in respect to their workload. Therefore, the period of data collection was rather long, and the numbers of applications remained low, which raised questions on the data saturation and which limited the generalisation of the results of this study. However, a certain amount of data saturation seemed reached and the limitation forced the researchers to analyse the available material more thoroughly. Analysing the narratives from the in-depth interviews appeared suitable. Retrospectively, it is this combination of grounded theory building and narrative analysis that provided us with a comprehensive understanding of impact along the two dimensions, of health decline and social network change, in older adults who are ageing in place. Another strength of the current study was that the open interviews with older adults themselves enabled us to analyse the voices of the ones profoundly at stake.

## Conclusion

This study aimed to explore experiences of social network change and health decline and the impact on older adults. Results indicate that health decline and social network change are unavoidable and uncontrollable (temporary) stressors that might, but not necessarily, hinder successful ageing. We distinguished four types of impact on older adults: A. Sneak preview of old age, B. Disruptive transition into old age, C. Drastically ageing, and D. Steadily ageing. The four types indicate that impact might diverge despite similar experiences, but also that older adults should be able to move along the four types of impact. These results present an optimistic view on the possibilities of older adults to continue ageing in place despite experiencing unavoidable and uncontrollable adversities in life. Also, the results provide leads for practice, to develop an action perspective for home care nurses and gerontological social workers to determine and reduce the impact of social network change and health decline on older adults who are ageing in place. Suggestions for further research would be to unravel how to detect temporal setbacks in successful ageing.

## Supplementary Information

**Additional file 1.**

**Additional file 2.**

## Data Availability

The datasets used and/or analysed during the current study are available from the corresponding author on reasonable request.

## References

[1] Wiles JL, Leibing A, Guberman N, Reeve J, Allen RES (2012). The meaning of “aging in place” to older people. The Gerontologist.

[2] Kendig H, Gong CH, Cannon L, Browning C (2017). Preferences and predictors of aging in place: longitudinal evidence from Melbourne, Australia. J Hous Elder.

[3] Lum TYS (2014). Neighborhood support and aging-in-place preference among low-income elderly Chinese City-dwellers. J Gerontol B.

[4] Lin Y-Y, Huang C-S (2015). Aging in Taiwan: building a Society for Active Aging and Aging in place. Gerontologist.

[5] Jiang N, Lou VWQ, Lu N (2018). Does social capital influence preferences for aging in place? Evidence from urban China. Aging Ment Health.

[6] Pacolet, J., R. Bouten, and K. Versieck, Social protection for dependency in old age: a study of the fifteen EU member states and Norway. 2018: Routledge.

[7] Campen, C., et al., Met zorg ouder worden. (Webversion). 2013.

[8] Burt RS (1997). A note on social capital and network content. Soc Networks.

[9] Kahn, R.L. and T.C. Antonucci, Convoys of social support: A life-course approach. Aging: Social change, 1981: p. 383–405.

[10] Berkman LF, Glass T, Brissette I, Seeman TE (2000). From social integration to health: Durkheim in the new millennium. Soc Sci Med.

[11] Cohen S, Wills TA (1985). Stress, social support, and the buffering hypothesis. Psychol Bull.

[12] Cornwell B, Laumann EO (2015). The health benefits of network growth: new evidence from a national survey of older adults. Soc Sci Med.

[13] Freund AM, Baltes PB (1998). Selection, optimization, and compensation as strategies of life management: correlations with subjective indicators of successful aging. Psychol Aging.

[14] Huber M, van Vliet M, Giezenberg M, Winkens B, Heerkens Y, Dagnelie PC, Knottnerus JA (2016). Towards a ‘patient-centred’operationalisation of the new dynamic concept of health: a mixed methods study. BMJ Open.

[15] Jowkar B, Friborg O, Hjemdal O (2010). Cross-cultural validation of the resilience scale for adults (RSA) in Iran. Scand J Psychol.

[16] Kawachi I (2001). Social Capital for Health and Human Development. Development.

[17] Seeman TE, Lusignolo TM, Albert M, Berkman L (2001). Social relationships, social support, and patterns of cognitive aging in healthy, high-functioning older adults: MacArthur studies of successful aging. Health Psychol.

[18] Uchino BN, Cacioppo JT, Kiecolt-Glaser JK (1996). The relationship between social support and physiological processes: a review with emphasis on underlying mechanisms and implications for health. Psychol Bull.

[19] Park S, Smith J, Dunkle RE (2014). Social network types and well-being among south Korean older adults. Aging Ment Health.

[20] Park NS, Jang Y, Lee BS, Chiriboga DA, Chang S, Kim SY (2018). Associations of a social network typology with physical and mental health risks among older adults in South Korea. Aging Ment Health.

[21] Jones PS, Lee JW, Zhang XE (2011). Clarifying and measuring filial concepts across five cultural groups. Res Nurs Health.

[22] Luppa M, Luck T, Weyerer S, König HH, Brähler E, Riedel-Heller SG (2009). Prediction of institutionalization in the elderly. A systematic review. Age Ageing.

[23] Nihtilä EK, Martikainen PT, Koskinen SV, Reunanen AR, Noro AM, Häkkinen UT (2007). Chronic conditions and the risk of long-term institutionalization among older people. Eur J Pub Health.

[24] Andel R, Hyer K, Slack A (2007). Risk factors for nursing home placement in older adults with and without dementia. J Aging Health.

[25] Broese van Groenou M, Hoogendijk EO, van Tilburg TG (2013). Continued and new personal relationships in later life:differential effects of health. J Aging Health.

[26] Aartsen MJ, van Tilburg T, Smits CHM, Knipscheer KCPM (2004). A longitudinal study of the impact of physical and cognitive decline on the personal network in old age. J Soc Pers Relat.

[27] Suanet B, van Tilburg TG, Broese MI (2013). Van Groenou, nonkin in older adults' personal networks: more important among later cohorts? The journals of gerontology. B Psychol Sci Soc Sci.

[28] Van Houtven CH, Norton EC (2004). Informal care and health care use of older adults. J Health Econ.

[29] Marengoni A, Angleman S, Melis R, Mangialasche F, Karp A, Garmen A, Meinow B, Fratiglioni L (2011). Aging with multimorbidity: a systematic review of the literature. Ageing Res Rev.

[30] Péres K (2008). Natural history of decline in instrumental activities of daily living performance over the 10 years preceding the clinical diagnosis of dementia: a prospective population-based study. J Am Geriatr Soc.

[31] Baltes PB, Baltes MM (1990). Psychological perspectives on successful aging: the model of selective optimization with compensation. Successful Aging Perspect Behav Sci.

[32] Carstensen LL (1992). Social and emotional patterns in adulthood: support for socioemotional selectivity theory. Psychol Aging.

[33] Charles ST, Piazza JR (2009). Age differences in affective well-being: context matters. Soc Personal Psychol Compass.

[34] Charles ST, Carstensen LL (2010). Social and emotional aging. Annu Rev Psychol.

[35] English T, Carstensen LL (2014). Selective narrowing of social networks across adulthood is associated with improved emotional experience in daily life. Int J Behav Dev.

[36] Krause N (2004). Lifetime trauma, emotional support, and life satisfaction among older adults. The Gerontologist.

[37] Shaw BA, Krause N, Liang J, Bennett J (2007). Tracking changes in social relations throughout late life. J Gerontol Ser B Psychol Sci Soc Sci.

[38] Carstensen LL (2006). The influence of a sense of time on human development. Science.

[39] Sims T, Hogan CL, Carstensen LL (2015). Selectivity as an emotion regulation strategy: lessons from older adults. Curr Opin Psychol.

[40] Kallio H, Pietilä AM, Johnson M, Kangasniemi M (2016). Systematic methodological review: developing a framework for a qualitative semi-structured interview guide. J Adv Nurs.

[41] Allmark P, Boote J, Chambers E, Clarke A, McDonnell A, Thompson A, Tod AM (2009). Ethical issues in the use of in-depth interviews: literature review and discussion. Res Ethics.

[42] O’Brien BC, Harris IB, Beckman TJ, Reed DA, Cook DA (2014). Standards for reporting qualitative research: a synthesis of recommendations. Acad Med.

[43] Luijkx K, van Boekel L, Janssen M, Verbiest M, Stoop A (2020). The academic collaborative center older adults: a description of co-creation between science, care practice and education with the aim to contribute to person-centered Care for Older Adults. Int J Environ Res Public Health.

[44] Smith PF, Larkin M (2009). Interpretative phenomenological analysis: theory, method and research.

[45] Corbin J, Strauss AL (2014). Basics of qualitative research.

[46] Langley A (1999). Strategies for theorizing from process data. Acad Manag Rev.

[47] Doucet A, Mauthner NS (2008). What can be known and how? Narrated subjects and the listening guide. Qual Res.

[48] Forrest J, Nikodemos L, Gilligan C (2016). The experience of receiving scholarship aid and its effect on future giving: a listening guide analysis. Qual Res Psychol.

[49] Jonker AAGC, Comijs HC, Knipscheer KCPM, Deeg DJH (2009). The role of coping resources on change in well-being during persistent health decline. J Aging Health.

[50] Hoyt MA, Wang AWT, Boggero IA, Eisenlohr-Moul TA, Stanton AL, Segerstrom SC (2020). Emotional approach coping in older adults as predictor of physical and mental health. Psychol Aging.

[51] Kovacs B (2021). Social networks and loneliness during the COVID-19 pandemic. Socius.

[52] Krendl AC, Perry BL (2020). The impact of sheltering in place during the COVID-19 pandemic on older adults’ social and mental well-being. J Gerontol B.

[53] Marroquín B, Vine V, Morgan R (2020). Mental health during the COVID-19 pandemic: effects of stay-at-home policies, social distancing behavior, and social resources. Psychiatry Res.

[54] Kivi M, Hansson I, Bjälkebring P (2020). Up and about: older adults’ well-being during the COVID-19 pandemic in a Swedish longitudinal study. J Gerontol B.

[55] Wells M (2012). Resilience in older adults living in rural, suburban, and urban areas. Online J Rural Nurs Health Care.

[56] Gooding PA, Hurst A, Johnson J, Tarrier N (2012). Psychological resilience in young and older adults. Int J Geriatr Psychiatry.

[57] MacLeod S, Musich S, Hawkins K, Alsgaard K, Wicker ER (2016). The impact of resilience among older adults. Geriatr Nurs.

[58] Allen J, Hutchinson AM, Brown R, Livingston PM (2017). User experience and care integration in transitional Care for Older People from Hospital to home:a meta-synthesis. Qual Health Res.

[59] Ten Bruggencate T, Luijkx KG, Sturm J. Social needs of older people: a systematic literature review. Ageing Soc. 2017:1–26.

[60] Machielse A (2015). The heterogeneity of socially isolated older adults: a social isolation typology. J Gerontol Soc Work.

[61] Cloutier-Fisher D, Kobayashi K, Smith A (2011). The subjective dimension of social isolation: a qualitative investigation of older adults' experiences in small social support networks. J Aging Stud.

[62] Kok AAL (2015). Capturing the diversity of successful aging: an operational definition based on 16-year trajectories of functioning. The Gerontologist.

[63] Smith JL, Hollinger-Smith L (2015). Savoring, resilience, and psychological well-being in older adults. Aging Ment Health.

[64] Ray M (2014). Gerontological social work: reflections on its role, purpose and value. Br J Soc Work.

