# Supplementary material for: The impact of social network change and health decline: a qualitative study on experiences of older adults who are ageing in place
Source: BMC Geriatr. 2021 Sep 4;21:480. doi: 10.1186/s12877-021-02385-6 (PMC8418744; doi:10.1186/s12877-021-02385-6)
Supplement: Supplementary file 2 — Additional file 2. [file 12877_2021_2385_MOESM2_ESM.pdf]

Code	Code Group 1	Code Group 2	Code Group 3
Aanpassen aan veranderingen lukt niet meer goed			
Acceptatie/berusting/knop omzetten	Actie		
Achtergelaten			
Actieradius is kleiner (groter) geworden			
Afhankelijk			
Afscheid nemen			
'Alleen'-gevoel/'samen-sterk'			
Anderen niet belasten met ondersteuningsvraag			
Bang (wel/niet)		Emotie	
Begrip voor situatie	Actie		
Beschermd wonen is (niet) aantrekkelijk			
Bezig met overleven	Actie		Ervaring health decline 1: Temporarily losing yourself
Blij (of niet) met ontvangen ondersteuning			
Blijven meedoen			
Boos		Emotie	
Buiten/afhankelijk van het weer (meer/minder)			
Contact met arts (nauwelijks, goed)			
Contact met familie			
Contact met paramedici			
Contact met V&V			
Contact met vrienden			
Contact woonomgeving			
Continu beschikbaar voor ondersteuning			
Cultuurminnend			
Dag en nacht			
Diagnose			
Direct na behandeling naar huis			
Echtgenoten zijn/doen verschillend			
Een leven lang samen			
Ergens nodig zijn			
Ervaring met ziekenhuisopnames			
Fijn om contact van mens tot mens te hebben met arts			
Fijn om thuis te zijn			
Frisse start/Nieuwe start			
Frustratie			
Fysiek is minder (conditie, lich. functies)			
Gaat graag uit			
Gastvrij			
Gebeurtenis 'middle aged'			
Gebeurtenis 'present'			
Gebeurtenis 'recent history'			
Gebeurtenis 'young'			
Gebeurtenis 'young old'			
Gebreken die al aanwezig waren komen nu meer op de voorgrond			
Geen angstig type			
Geen/wel 100% herstel			
Gemis			
Gemis daginvulling (of juist niet)			

Geschrokken			
Gevoelsmatig klaar met herstellen			
Goed in staat om hulpvraag te formuleren			
Goed mens			
Herinneringen			
Het gaat niet meer			
Hoeveelheid contacten (omvang netwerk)			
Hulpmiddel			
Iets terug willen doen			
IJzeren regelmaat/gewoon doorgaan	Actie		
Impact (nauwelijks/veel)			
In de steek gelaten door professionals			
Intensiteit sociale contacten groter/kleiner (rondom zkh opname/na achteruitgang)			
Je bent jezelf niet meer of juist nog wel (medicatie, gebeurtenissen, ziekte)			
Kan zichzelf aardig redden			
Keihard			
Keuze voor wonen (in stad wegens voorzieningen/elders wegens kinderen)			
Klachten van kwaad tot erger			
Lage/hoge SES			
Langer verblijf in ziekenhuis			
Langlopend/sinds kort contact			
Lastig om eigen grenzen te bewaken/zoekt grenzen op			
Leefstijl (eco, gezelligste buurt, boer)			
Levenseinde			
Maatschappelijk actief			
Machteloosheid		Emotie	
Mantelzorg			
Medische betrokkenheid is vervallen			
Meer nodig			
Moe			
Moeite met contacten leggen			
Mooi			
Na afloop van herstel/dip/overlijden			
Niet eenzaam			
Niet gemakkelijk om thuis patient te zijn			
Niet meer dezelfde persoon (eigenschappen)			
Niet/wel in staat om hulpvraag te formuleren	Actie		Ervaring health decline 1: Temporarily losing yourself
Nieuwe orde creeren kost veel tijd en energie			
Ondersteuning door familie			
Ondersteuning door professionals (wel/niet)			
Ondersteuning door sociaal netwerk			
Ondersteuning door woonomgeving			
Onduidelijkheid over medische toestand			
Ongemerkt (sluipt er in)/kink in de kabel			
Ongerust			
Onvergetelijk			
Onzekerheid (over herstel)			
Opgelucht		Emotie	
Opname in hospice			

Opname in zorghotel/revalidatielocatie			
Oppervlakkig/diepgaand/lotgenoten contact			
Optimist/pessimist			
Ordentelijk			
'Oud' volgens anderen/zelf			
Overal in NL gewoond			
Overziet situaties minder goed			
Periode 'middle aged'			
Periode 'present'			
Periode 'recent history'			
Periode voor ziekenhuisopname			
Periode 'young'			
Periode 'young old'			
Pijn			
Praat gemakkelijk over wel en wee tegen anderen			
Pragmatisch			
Prettig (of niet)			
Professionals schatten capaciteiten patient te hoog in op moment van dip			
Recht voor z'n raap			
Regelt alles zelf ("Je moet het zelf doen")			
Relativeren van situatie met humor			
Respijt			
Rijk bestaan/Blij (Treuren) met veel (weinig) contacten			
Schaamte (of niet)		Emotie	
Scheiding door vph-opname			
Sociaal persoon			
Spijt			
Spirituele ervaring			
Spontaan aangeboden hulp			
Spontaan bezoek			
Spontaan ontmoeten			
Sportief			
Stabiel/labiel persoon			
Stapeling van mankementen			
Steeds minder sociale contacten/geen nieuwe			
Stress (of juist zonder)			
Teleurgesteld/verrast door sociale contacten tijdens/na ziekbed			
Tevreden/niet tevreden			
Thuis (redderen, weer gewoon)			
Thuis wonen zolang het nog gaat	Actie		
Trots			
Veel meegemaakt/Levenservaring			
Verbaasd			
Verdriet		Emotie	
Verontwaardigd		Emotie	
Verplaatsingen (zkh/rev/thuis) zijn zwaar			
Verrast			
Verschrikkelijk			
Versnipperd/1 loket			

Vertrouwen/wantrouwen			
Verwacht (geen) verder herstel/kijkt (niet meer) vooruit	Actie		
Voelt zich (niet) gehoord/gezien (door professionals, anderen)			
Volhouden/Opgeven	Actie		
Volledig van de wereld geweest			Ervaring health decline 1: Temporarily losing yourself
Vooraf zelf al ondersteuning geregeld	Actie		
Vooruitzicht onbekend			
Vrijheid (of het ontbreken daaraan)			
Waardering			
Wachten			
Woonomgeving met veel/weinig verbondenheid			
Ziekenhuisopname			
Zwaar			

Code Group 4	Code Group 5	Code Group 6
		Ervaring health decline 4: Being aware of permanent setback
	Ervaring health decline 3: Home (bitter) sweet home	
	Ervaring health decline 3: Home (bitter) sweet home	
Ervaring health decline 2: preferring medical attention		
Ervaring health decline 2: preferring medical attention		
	Ervaring health decline 3: Home (bitter) sweet home	
		Ervaring health decline 4: Being aware of permanent setback
		Ervaring health decline 4: Being aware of permanent setback

Ervaring health decline 2: preferring medical attention		
		Ervaring health decline 4: Being aware of permanent setback
Ervaring health decline 2: preferring medical attention		
Ervaring health decline 2: preferring medical attention		
	Ervaring health decline 3: Home (bitter) sweet home	
		Ervaring health decline 4: Being aware of permanent setback
	Ervaring health decline 3: Home (bitter) sweet home	
	Ervaring health decline 3: Home (bitter) sweet home	
	Ervaring health decline 3: Home (bitter) sweet home	
	Ervaring health decline 3: Home (bitter) sweet home	
	Ervaring health decline 3: Home (bitter) sweet home	
Ervaring health decline 2: preferring medical attention		

		Ervaring health decline 4: Being aware of permanent setback
Ervaring health decline 2: preferring medical attention		
	Ervaring health decline 3: Home (bitter) sweet home	
	Ervaring health decline 3: Home (bitter) sweet home	

		Ervaring health decline 4: Being aware of permanent setback
Ervaring health decline 2: preferring medical attention		

Code Group 7	Code Group 8	Code Group 9
Ervaring health decline 5: Adapting oneself and continuing life		
Ervaring health decline 5: Adapting oneself and continuing life		
Ervaring health decline 5: Adapting oneself and continuing life		
	Ervaring SN change 1: Regretting social losses	
	Ervaring SN change 1: Regretting social losses	
	Ervaring SN change 1: Regretting social losses	
		Ervaring SN change 2: Gratitude for support

Ervaring health decline 5: Adapting oneself and continuing life		
		Ervaring SN change 2: Gratitude for support
		Ervaring SN change 2: Gratitude for support
Ervaring health decline 5: Adapting oneself and continuing life		
Ervaring health decline 5: Adapting oneself and continuing life		
		Ervaring SN change 2: Gratitude for support
Ervaring health decline 5: Adapting oneself and continuing life		

Ervaring health decline 5: Adapting oneself and continuing life		
Ervaring health decline 5: Adapting oneself and continuing life		
	Ervaring SN change 1: Regretting social losses	
	Ervaring SN change 1: Regretting social losses	
	Ervaring SN change 1: Regretting social losses	

		Ervaring SN change 2: Gratitude for support
	Ervaring SN change 1: Regretting social losses	

Code Group 10	Code Group 11	Code Group 12	Code Group 13	Code Group 14	Code Group 15
					Kenmerk gezondheid
	Ervaringen				
					Kenmerk gezondheid
	Ervaringen				
	Ervaringen				
	Ervaringen				
	Ervaringen				
	Ervaringen				
Ervaring SN change 3: Persisting social engagement					
				Kenmerk fysieke omgeving	
	Ervaringen				
	Ervaringen				
	Ervaringen				
	Ervaringen				
	Ervaringen				
	Ervaringen				
	Ervaringen				
	Ervaringen				
	Ervaringen				
	Ervaringen				
	Ervaringen				
	Ervaringen				
	Ervaringen				
		Fase in verandertraject/leven			
	Ervaringen				
					Kenmerk gezondheid
Ervaring SN change 3: Persisting social engagement					
Ervaring SN change 3: Persisting social engagement					
			Gebeurtenissen van belang		
			Gebeurtenissen van belang		
			Gebeurtenissen van belang		
			Gebeurtenissen van belang		
			Gebeurtenissen van belang		
					Kenmerk gezondheid
					Kenmerk gezondheid
	Ervaringen				
	Ervaringen				

	Ervaringen				
	Ervaringen				
	Ervaringen				
	Ervaringen				
	Ervaringen				
				Kenmerk fysieke omgeving	
	Ervaringen				
	Ervaringen				
	Ervaringen				
				Kenmerk fysieke omgeving	
					Kenmerk gezondheid
				Kenmerk fysieke omgeving	
	Ervaringen				
		Fase in verandertraject/leven			
	Ervaringen				
	Ervaringen				
	Ervaringen				
		Fase in verandertraject/leven			
	Ervaringen				
	Ervaringen				
	Ervaringen				
	Ervaringen				
	Ervaringen				
	Ervaringen				
	Ervaringen				
	Ervaringen				

		Fase in verandertraject/leven			
				Kenmerk fysieke omgeving	
					Kenmerk gezondheid
		Fase in verandertraject/leven			
		Fase in verandertraject/leven			
		Fase in verandertraject/leven			
		Fase in verandertraject/leven			
		Fase in verandertraject/leven			
		Fase in verandertraject/leven			
	Ervaringen				
	Ervaringen				
	Ervaringen				
Ervaring SN change 3: Persisting social engagement					
	Ervaringen				
Ervaring SN change 3: Persisting social engagement					
					Kenmerk gezondheid
	Ervaringen				
	Ervaringen				
	Ervaringen				
		Fase in verandertraject/leven			
	Ervaringen				
	Ervaringen				
	Ervaringen				
	Ervaringen				
	Ervaringen				

	Ervaringen				
	Ervaringen				
					Kenmerk gezondheid
	Ervaringen				
	Ervaringen				
		Fase in verandertraject/leven			
	Ervaringen				

Code Group 16	Code Group 17	Code Group 18
		Kenmerk van relatie tussen persoon en omgeving
Kenmerk persoon		
		Kenmerk van relatie tussen persoon en omgeving
Kenmerk persoon		
Kenmerk persoon		
Kenmerk persoon		
Kenmerk persoon		
Kenmerk persoon		

Kenmerk persoon		
	Kenmerk sociale omgeving	
		Kenmerk van relatie tussen persoon en omgeving
		Kenmerk van relatie tussen persoon en omgeving
Kenmerk persoon		
Kenmerk persoon		
	Kenmerk sociale omgeving	
		Kenmerk van relatie tussen persoon en omgeving
Kenmerk persoon		
		Kenmerk van relatie tussen persoon en omgeving
		Kenmerk van relatie tussen persoon en omgeving
	Kenmerk sociale omgeving	
Kenmerk persoon		
Kenmerk persoon		
		Kenmerk van relatie tussen persoon en omgeving
		Kenmerk van relatie tussen persoon en omgeving
		Kenmerk van relatie tussen persoon en omgeving
		Kenmerk van relatie tussen persoon en omgeving

		Kenmerk van relatie tussen persoon en omgeving
Kenmerk persoon		
Kenmerk persoon		
		Kenmerk van relatie tussen persoon en omgeving
Kenmerk persoon		
Kenmerk persoon		
		Kenmerk van relatie tussen persoon en omgeving
Kenmerk persoon		
Kenmerk persoon		
Kenmerk persoon		
	Kenmerk sociale omgeving	
		Kenmerk van relatie tussen persoon en omgeving
Kenmerk persoon		
		Kenmerk van relatie tussen persoon en omgeving
		Kenmerk van relatie tussen persoon en omgeving
		Kenmerk van relatie tussen persoon en omgeving
Kenmerk persoon		
Kenmerk persoon		
	Kenmerk sociale omgeving	
Kenmerk persoon		
	Kenmerk sociale omgeving	

		Kenmerk van relatie tussen persoon en omgeving
	Kenmerk sociale omgeving	
